# Recommendations for the application and follow-up of quality controls in medical laboratories

**DOI:** 10.11613/BM.2021.020501

**Published:** 2021-04-15

**Authors:** Jean-Marc Giannoli, Stéphanie Albarede, Thierry Avellan, Jean-Pierre Bouilloux, Régine Cartier, Richard Cohen, Nathalie Colard, Luc Essemilaire, Jean-Louis Galinier, Mathieu Kuentz, Mickaël Paris, Henri Portugal, Florian Scherrer, Jean-Pascal Siest, Anne Vassault, Jean-Michel Vialle

**Affiliations:** 1 Accredited Medical Laboratories Network (LABAC), Lyon, France; 2 Toulouse Center for Quality Control in Medical laboratories (CTCB), Toulouse, France; 3 Association for Promotion of Quality Control in Medical Laboratories (Probioqual), Lyon, France; 4 French Society for Medical Biology (SFBC), Paris, France; 5 Biologie Prospective, Villers-lès-Nancy, France; 6 Quality Assurance for Medical Laboratories (Asqualab), Paris, France

**Keywords:** risk analysis, internal quality control, external quality assessment, uncertainty

## Abstract

This is a translation of the paper “Recommendations for the application and follow-up of quality controls in medical biology laboratories” published in French in the journal Annales de Biologie Clinique (Recommandations pour la mise en place et le suivi des contrôles de qualité dans les laboratoires de biologie médicale. Ann Biol Clin (Paris). 2019;77:577-97.). The recommendations proposed in this document are the result of work conducted jointly by the Network of Accredited Medical Laboratories (LABAC), the French Society of Medical Biology (SFBC) and the Federation of Associations for External Quality Assessment (FAEEQ). The different steps of the implementation of quality controls, based on a risk analysis, are described. The changes of reagent or internal quality control (IQC) materials batches, the action to be taken in case of non-conform IQC results, the choice of external quality assessment (EQA) scheme and interpretation of their results as well as the new issue of analyses performed on several automatic systems available in the same laboratory are discussed. Finally, the concept of measurement uncertainty, the robustness of the methods as well as the specificities of near-patient testing and rapid tests are described. These recommendations cannot apply for all cases we can find in medical laboratories. The implementation of an objective alternative strategy, supported with documented evidence, might be equally considered.

## Introduction

This is a translation of the paper “Recommendations for the application and follow-up of quality controls in medical biology laboratories” published in French in the journal Annales de Biologie Clinique (Recommandations pour la mise en place et le suivi des contrôles de qualité dans les laboratoires de biologie médicale. Ann Biol Clin (Paris). 2019;77:577-97.) (*1*).

The recommendations proposed in this document come mainly from the conference jointly organized by the Network of Accredited Medical Laboratories (LABAC), the French Society of Medical Biology (SFBC) and the Federation of Associations for External Quality Assessment (FAEEQ) in Paris on January 30th, 2019. This conference was an opportunity to discuss internal quality control (IQC), external quality assessment (EQA) practices and estimation of measurement uncertainties (MU) for routine quantitative methods (biochemistry, haematology and haemostasis). Microbiology testing (including infectious serology) is excluded from the scope of these recommendations, as well as specialized tests where quality control practices should be adapted.

The various contributors have endeavoured to establish explicit positions that reflect literature, experience and knowledge in the field of medical laboratories. These recommendations are designed to be considered as a basis for reflection and work practices for all those involved in medical laboratories. These recommendations do not pretend to respond to every conceivable situation within a laboratory. The implementation of an alternative strategy that is well argued and objective is also considered. Finally, these recommendations are naturally likely to evolve over time.

Three levels of recommendations were proposed (*2*):

Recommended practices (deemed to comply with the requirements of standard ISO 15189:2012 (*3*)): These are derived from reference documents, consensus data from various publications or may also be based on at least one publication with robust methodology and interpretation criteria (expert opinion). They represent best practice, the “state of the art”. They are considered good practice objectives.
Acceptable practices: These are established taking into account the various bibliographical data that have been subject to varying interpretations in different publications or, failing that, a publication for which the interpretation criteria are not as strong as in the recommended category (*e.g.* fewer authors, statistical methodology used, *etc.*).
Inappropriate practices (deemed not to comply with the requirements of the ISO standard 15189:2012): Unacceptable practice for which there is a consensus after reading the different publications or a defect based on at least one publication for which the methodology and interpretation criteria are sound and robust (expert opinion). Such inappropriate practices may compromise the reliability of the results.

With regard to IQC, the ISO 15189:2012 standard requires the following (*3*):

Design control procedures to ensure that the expected quality of results is achieved;
Use control materials that behave as similar as possible with patient samples (notion of commutability);
Periodically analyse control materials according to the stability of the system;
In case of non-conformity, assess the impact on any results already reported since the last acceptable IQC;
Regularly review IQC results to detect drifts and trends.

The SH REF 02 rev. 05 document reports the concept of a documented strategy including the definition of series, frequency of use, levels used, performance requirements and validation rules (*4*). It also deals with the measures to be taken in the event of a non-conformity, results that do not comply with the defined rules and the estimate of a possible impact on the results already reported.

ISO/IEC 17025:2017 standard introduces the concept of “ensuring the validity of results” (*5*).

## Risk analysis, the definition of series

### Risk analysis

Risk analysis is the essential first step in the implementation of an IQC strategy. It consists of a summary of analytical issues that could lead to a potentially erroneous result (Table 1). The list below is non-exhaustive and includes the main risks identified:

**Table 1:** Summary of analytical risks

**Risk**	**Identification of risk and potentially erroneous result**	**Recommendations: suppliers or best practices**	**Limitations of these recommendations**	**Effectiveness** **of correction**	**Other possible actions** **(master plan)**	**Residual risk**	**Indicators**
1	Deterioration of reagent during transport	- Separate shipment of reagents and control materials. Note: Third party controls are preferred. - Carry out inspections on receipt (5.3.2.3, standard ISO 15189:2012) according to the identification of high risks.	If the control is changed, no detection of the anomaly.	Partial	- Separate reagents and IQC materials when ordering - Monitor the storage of IQCs (temperature) and be aware of acceptance tests - Check the expiry date before and after the vials are opened. - Use multiple control levels (limit of quantification (LOQ)), threshold value, linearity limit) - Acceptable limits adapted to clinical needs	Acceptable	- Number of non-conformities at reception - Number of non-conformities due to inappropriate storage temperature - Non-conformities detected during the internal technical audit - IQC CV follow-up - Recording of EQA compliance and non-compliance and periodic review
2	Inappropriate calibration data	- Acceptable limits of the calibrator signal (software) - IQC post-calibration - Verification of calibration data with appropriate acceptable criteria.	Possible drift between 2 IQC results	Partial	- IQC before/after recalibration - Analyse multiple patient samples prior to calibration - Frequency strategy for performing IQC based on the number of analyses and the criticality of the analyte - Participation in EQA - Monitoring of temperature and temperature variations	Acceptable	- Number of calibration failures - Number of additional calibration as compared to the supplier specification - Number of calibrations outside the supplier’s specifications - IQC CV data - Conformity of EQA results
3	Micro clogging that totally or partially obstructs the system.	- Measurement of the pressure in the pipetting system (if available and if the analyser alarm exists) - Optical detection for checking the volume of the sample taken	Micro-clots are not detected.	Partial	- Visual inspection of samples - Monitor the frequency of clogging problems - IQC before and after maintenance (syringe exchange, decontamination...)	Acceptable	Number of non-conformities due to total or partial fouling
**Risk**	**Identification of risk and potentially erroneous result**	**Recommendations: suppliers or best practices**	**Limitations of these recommendations**	**Effectiveness** **of correction**	**Other possible actions** **(master plan)**	**Residual risk**	**Indicators**
4	Defective maintenance	- Recalibrate after maintenance	Lack of detection system of drift, corrected by recalibration	Partial	- IQC before and after maintenance (syringe exchange, decontamination...) - Analyse several patient samples that were analysed prior to the interview - Looking for highly sensitive surveillance tests -Control of maintenance operations in terms of system stability	Acceptable	- Number of IQCs rejected after maintenance - Number of maintenance errors - Non-conformities detected during an internal audit
5	Deterioration of the reagent during storage or use, use of expired reagents	- Expiration date control by barcode on the analyser - Software for signal error detection (baseline, DO calibration) - IQC	- No detection of the shelf life of open reagents (measurement of the delay between the analyser and storage)	Partial for open reagents	-Training, user qualification -Traceability of reagent openings -IQC for each series of tests - Monitor storage temperature	Acceptable	Non-conformities detected during the internal technical audit on the traceability of the use of reagents and controls
6	System failure (locking and non-locking)	- Corrective maintenance and IQC	- No detection of abnormalities prior to failure	Partial for failure blocking	- IQC after maintenance - Retesting patient samples after resolving the problem	Acceptable	- Number of patient reports recalled - Number of blocking failures
7	Uncontrolled environmental conditions (temperature drift over time)	- Definition of minimum/maximum temperature limits - Variation of the temperature between the calibration phase and the analysis phase and during the analysis phase	Lack of monitoring of temperature changes over time	Partial if variations are within acceptable temperature limits	- Traceability of temperature variations as a function of operations (calibration, IQC, patient samples) - Bracketed series by additional controls or tests on patient samples	Acceptable	- Use of temperature monitoring data during the analysis process - Monitoring of temperature control cards
**Risk**	**Identification of risk and potentially erroneous result**	**Recommendations: suppliers or best practices**	**Limitations of these recommendations**	**Effectiveness** **of correction**	**Other possible actions** **(master plan)**	**Residual risk**	**Indicators**
8	Deviations over time (drift and trend)	- IQC strategy by reagent and analyte	-Frequency too low -Range of acceptable limits too wide - Inadequate Westgard rules	Partial for drift detection	- IQC with acceptable limits adapted to the actual performance of the analyzer and clinical needs - Visual assessment of drift and trend control charts - Monitoring of CVs according to relevant specifications - 6 Sigma calculation - Monitoring of patient mean values - If several analysers, check for comparability - Monitor bias by comparing it to a peer group	Acceptable	- Long-term CV monitoring by analyzer - Conformity of CVs with laboratory objectives
9	Operator error (manual techniques)	- Authorization of qualification (skills assessment)	- Diversion of practices	Partial	- Appropriate frequency of authorizations - Audit with observation of practices (inter-operator variability) - Checking manual entry	Acceptable	- Monitoring of IQC and EQA per operator
10	Operator error (automatic techniques)	- Password changed frequently - Special access code for the administrator to change the configuration of scans and controls	- Diversion of practices	Partial	- Password security - Appropriate frequency of authorizations	Acceptable	- IT security audit - Announces changes in the configuration of analyses and controls
IQC – internal quality control. EQA – external quality assessment. CV – coefficient of variation. DO – optical density. IT- information technology.

1. Reagent defect during shipment
2. Sample abnormalities
3. Presence of micro clots totally or partially obstructing the pipetting system
4. Faulty or insufficient maintenance
5. Deterioration of the reagent during storage in the laboratory (lack of stability of the reagent) or expiration of the reagent
6. Blocking and non-blocking anomaly or drift of the analytical system
7. Uncontrolled environmental conditions (temperature and variation of temperature over time, humidity, *etc.*)
8. Drift over time of the method (drift and trend)
9. Error by operator effect (manual methods)
10. Error by operator effect (automatic methods).

These risks depend on each method and are, for the most part, identified. Suppliers of *in vitro* diagnostic medical devices provide control materials. However, these recommendations may be insufficient or inappropriate and further action may be required. All of these actions should be associated with indicators that make possible to monitor risk control by recording situations where the risk is no longer under control (*e.g.*, monitoring non-conformities). As part of a continuous improvement approach, a reassessment of the risk (with new input data: non-conformity, internal and external audits, complaints, indicators, *etc.*) is necessary with the implementation of any new means of control. This “dynamic” approach allows for a pro-active system.

Other risks may be identified, depending in particular on the analyser or method used (*e.g.* quality of consumables, water quality, *etc.*). It is the laboratory’s responsibility to set up appropriate control resources. The risks monitored by IQCs correspond to 1, 2, 3, 4, 5, 6, 7, 8, 9 (Table 2).

**Table 2:** Risk analysis

Recommended practice	A risk analysis that defines means of control for all identified risks associated with dynamic monitoring of the risk, assessment of risk control and its dynamic monitoring.
Acceptable practice	Risks controlled but not evaluated (not practice monitored and incomplete dynamic follow-up) and without impact on the patient.
Unacceptable practice	Lack of formal risk analysis and/or management.

### Study of the robustness of the method

Standard ISO 15189:2012 in 5.6.2.1 specifies that the expected quality of the results must be verified (*3*). Firstly, it is necessary to determine the robustness of the method (Table 3).

**Table 3:** Study of the robustness of the method

Recommended practice	Sigma calculation documenting the choice of total allowable error. Use of other means to assess robustness. Compare with other indicators (monitoring of IQCs, EQAs, *etc.*). Adjustment of series size, IQC frequency and Westgard rules with respect to Sigma data.
Acceptable practice	The level of sigma or other means of assessing robustness used only as an indicator. Lack of specific robustness assessment but management strategy for an adapted IQC (no impact on the patient).
Unacceptable practice	Does not consider the robustness of the method.
IQC – internal quality control. EQA – external quality assessment.	

The Six Sigma approach (Sigma = (TE_A_ - Bias)/CV) is not an objective in itself but a tool for assessing the robustness of the method (*6*). The sigma level is calculated from the total allowable error (TE_A_) chosen by the laboratory, coefficients of variation (CVs) and biases that are objective data, characteristic of the method. The difficulty lies in the choice of the TE_A_, which can considerably modify the result of the Sigma level (*7*). There is currently a debate on the sigma calculation formula taking into account the bias (*8*).

Other means can be used to evaluate the robustness of a method: the repeatability/reproducibility ratio, frequency of recalibrations, frequency of re-targeting of IQCs mean, *etc.* (*5*).

The laboratory may also use the results of peer group methods (as trueness approach), through EQA survey reports and possibly the results of comparisons with other laboratories.

The Sigma calculation is mainly used to define the IQCs strategy as frequency of QC materials assays (Table 3) (*9*). The Six Sigma approach is suitable for large series.

### Strategy for frequency of IQC materials: definition of the series and critical events

The laboratory has to identify what is likely to affect the stability of the method process. The laboratory identifies critical events or critical control points (critical control point quality control): calibration, maintenance with direct impact on results (replacement of parts, adjustments, *etc.*), some types of failures, a change of reagents batch or calibrators, *etc.* The laboratory can thus define the events that will end the series (*9*).

The IQC’s scheduling strategy is based on (Table 4):

**Table 4:** Definition of the series and critical events

Recommended practice	Definition of critical events by the analyser and analysis to define the series and adapted IQC schedule.
Acceptable practice	Set the series in square brackets correctly, practice but no definition of critical points. Release of results prior to the results of “end-of-run” IQC (or other means of control of the method) and action to be taken if end-of-run IQC (or other means of control) are not in compliance. This strategy is defined according to the risk analysis of each analysis.
Unacceptable practice	No justification and explanation of the IQC practice schedule.
IQC – internal quality control.

the definition of the frequency of IQC materials: risks 2 and 5;
the levels used: risks 1 and 2;
positioning in the series (calibration, number of dosages): risks 2 and 7;
events likely to have an impact: risks 2, 3, 4, 6;
the definition of acceptable limits and the choice of rules for interpreting control charts: risks 1, 2, 8.

For the definition of the series, the following should also be taken into account:

stability of the sample (risk analysis to be carried out when the stability of the analyte does not always allow re-testing of the sample, *e.g.* bicarbonates);
the criticality of the test if the result is given in an emergency (troponin, D-Dimer, complete blood count, *etc*.) before an IQC is performed to close the series;
the robustness of the technique used;
manufacturer recommendations.

The laboratory therefore frames the series with IQCs or other means (Table 4). Nevertheless, “in the end”, after the last IQC, it is acceptable to consider that the method is under control and stable for a relatively short time still to be defined by the laboratory (a few hours according to the authors experience), for a limited number of tests (less than 50, according to the authors experience) taking into account the robustness of the method.

### Position of preventive maintenance in the IQCs schedule

#### See risk 4

Some preventive maintenance can have an impact on the method (to be documented with the supplier): it is important to consider this information to control your equipment.

The simplest way to monitor the impact of these maintenance activities on the stability of the system is to perform IQCs before and after maintenance activities, but the laboratory can also use other means (re-testing of samples, *etc*.) (Table 5).

**Table 5:** Position of the preventive maintenance

Recommended practice	Maintenance operations that have an impact on practice are identified. IQCs (or other means) are used before and after maintenance. Quality indicators are set up to control risk management.
Acceptable practice	Concepts are known and used, but not formalized in regular documentation (documents found but not included in the guidelines). Risk controlled by IQC test or other appropriate means.
Unacceptable practice	No knowledge of the nature of maintenance or its impact. No IQC used (or other means) before and after an impacting interview.
IQC – internal quality control.	

### Position of the curative maintenance

#### See risk 6

Curative maintenance might have an impact and, in this case, it ends a series unexpectedly. The laboratory must verify that there was no drift prior to failure (impact study) (Table 6).

**Table 6:** Position of curative maintenance

Recommended practice	The laboratory must verify by a method of its choice the absence of drift before failure: re-testing of patient samples, use of mean values of results, or any other means that must be justified. In case of re-testing of samples, the number depends on the size of the series (N) (square root of N, 10%, “rolling back the series” to determine when the system malfunction had an impact on the results, *etc.*). The impact study is recorded and corrective measures are implemented.
Acceptable practice	Review of all patient samples (subject to measurand stability) since the last valid IQC (or other relevant practice), but no reflection or strategy.
Unacceptable practice	No impact study and therefore potential release of erroneous results.
IQC – internal quality control.	

Number of tests in the series and frequency of IQCs

#### See risk 8

The laboratory must determine the frequency of IQCs and the series size (number of patient sample analyses for an analyte between two IQCs) (Table 7). The sigma level is one way (Table 4) to assess the robustness of the method, but other elements must also be taken into account in a risk analysis:

**Table 7:** Frequency of IQC

Recommended practice	A complete risk analysis must be carried out and an appropriate strategy defined, taking into account the Sigma level (or another relevant method to assess the robustness of the method) as well as Westgard rules. This strategy is adapted to each analyte.
Acceptable practice	No differentiation by analyte but satisfactory control of the risk of drift over time. Publication of results prior to the execution of “end-of-series” IQCs for critical tests involving risk to the patient, with documented risk analysis
Unacceptable practice	IQC frequency does not take into account method performance, criticality, and urgency of the test result.
IQC – internal quality control.

the clinical significance of the analyte
the time frame for the release and use of results
possibility of samples re-analysis (pre-analytical requirements applied), where applicable (impossible for some tests, such as blood gas analysis).

Some authors, in recent publications, propose to adapt the size of the series according to the Sigma level and the choice of Westgard rules (*10*-*12*).

### The choice of internal quality controls and the acceptable range

#### Standard requirements

##### See risks 1 and 2

The IQCs shall meet the following requirements of ISO 15189-2012 standard (5.6.2.2):

materials similar to patient samples as closely as possible. The non-commutability of IQCs is not prohibitive if they are more sensitive to analytical problems than patient samples (for intra-laboratory reproducibility monitoring);
a regular review of the results according to the stability of the method and the risk of impact on the patient care in the event of an erroneous result.
concentrations of control levels close to clinical decision levels.

### A manufacturer-independent IQC?

The ISO 15189:2012 standard, 5.6.2.2: note 2 (non opposable), recommends IQCs independent of the supplier to control a risk of non-detection of drift when changing reagent (poor compatibility or false alarm) (*3*). However, if the manufacturer is involved, the manufacturer’s IQC is required for critical analysis (Table 8).

**Table 8:** Choice of IQCs

Recommended practice	Use independent quality control instead of or in addition to supplier quality control.
Acceptable practice	Use only the IQCs provided by the reagent and/or analyser manufacturer.
Unacceptable practice	No IQC. Utilization of expired IQCs.
IQC – internal quality control.	

### Number and concentration levels

The number and concentration levels of IQCs also need to be defined: the IQC must explore the full range of measurements, but also the limits of the clinical decision. It shall also allow the calibration to be checked (Table 9).

**Table 9:** Number and concentration levels

Recommended practice	Use an IQC with level close to clinical decision limits practice (*e.g.,* infectious serology, troponin, D-Dimers, haemoglobin A1c, glucose). Cover the physiological and pathological range (if IQCs are available). Use multiple levels of IQC and at least 2 levels after calibration (12).
Acceptable practice	Other uses of IQC (excluding calibration verification).
Unacceptable practice	Use only one level of IQC after calibration practice (unless recommended by the supplier). No appropriate verification over the entire measuring range. Absence of a well thought-out strategy leading to a risk of not detecting drift of clinical decision limits in the measurement ranges.
IQC – internal quality control.	

### Acceptable limits

#### See risk 8

The main objective is to detect anomalies and trends (shift and drift) and to verify if the results achieve the required quality.

Acceptable limits (the term “analytical performance specification” is used in the latest European recommendations) should be selected based on the actual performance of the method to detect trends, shifts or deviations. Be careful not to confuse drift and shift. A drift is a constant increase (or decrease) in results. A shift corresponds to a constant deviation from the average.

The laboratory will choose its CVs for each analyte (see “expected quality” 5.6.2.1. of ISO 15189:2012) according to the performance of previous batches. These CVs are the laboratory’s own CVs: long-term CVs (CV_LT_) which are defined taking into account all sources of variability in the method. They are used to define acceptable limits for control charts.

In a second step in order to monitor the laboratory performance, it will be necessary to compare the CVs obtained with the reference, which might be:

CVs resulting from biological variations reported by the European Federation of Laboratory Medicine (EFLM) database as European Biological Variation Study (EuBIVAS);
CVs reported by the manufacturer;
CVs from peer groups (externalized IQCs);
CVs based on recommendations from French or foreign societies;
CVs from the recommendations of the EQA providers.

### In practice and when Westgard rules are used

The perspective presented in Table 10 allows the laboratory to ensure that analytical performance remains stable and under control. This proposal does not take into consideration the impact on clinical performance (or lack of impact) in case of unacceptable analytical performance (see Post-IQC impact study, end of series outside acceptable limits).

**Table 10:** Acceptable limits

Recommended practice	CV_LT_ used by the laboratory close to the long term CV of the method: the CV_LT_ will take into account changes in batches, operators, *etc.* (13).
Acceptable practice	Extended CV_LT_ (30% according to Fisher Snedecor without statistical significant difference) but allowing detection of trends and without exceeding the analytical objective or the maximum CV set by the laboratory for reproducibility during the verification of the method.
Unacceptable practice	The CV_LT_ does not detect trends. Control charts are not derived from laboratory data.
CV_LT_ – long-term coefficient of variation.	

An additional interpretation (at the end of the run) can be put in place to examine the potential clinical impact. Two levels of assessment are therefore in place: analytical acceptable limits and acceptable limits related to medical risk.

### Control rules

The objectives of the Westgard rules are as follows:

to detect a systematic or random analysis error
to stop publication of results in the event of a proven error
to estimate the bias (error) induced on previously published results in order to assess the impact on the patient and in terms of statistics, the objectives are to obtain:
a probability of error detection (PED) greater than 90%: this is a measure of the chance of detecting an error if there is a problem with the analysis method; this probability should be as high as possible.
a probability of false rejection (PFR) of less than 5%: this corresponds to the risk of rejecting a series in the absence of any problem with the analysis method; this probability should be as low as possible.

Some international recommendations allow choosing, as a minimum, the rejection rules without taking into account the robustness of the method (risk of over-quality) (Table 11) (*14*).

**Table 11:** Empirical multirole components rules for IQCs

**Rules (11)**		**Type of variability detected**
1_3S_		Imprecision or bias
2_2.5S_		Bias
R_4S_		Imprecision
8_1.5S_		Bias trend
IQC – internal quality control.		

**Figure 1:**
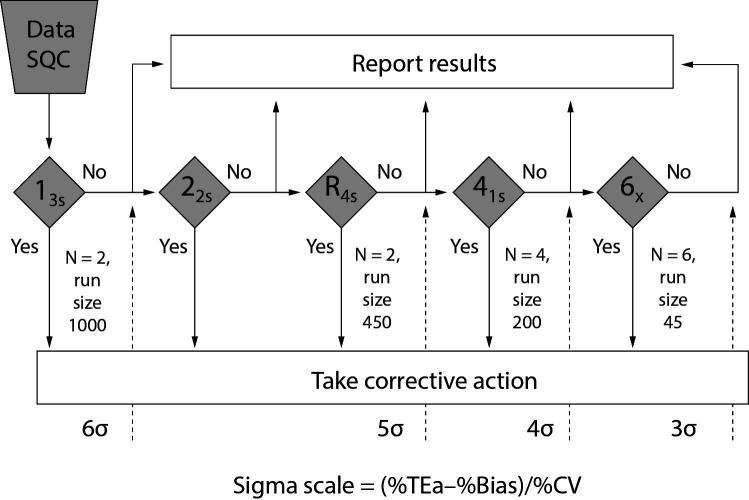
Westgard Sigma rules with run sizes for the numbers of patient samples between statistical quality control (SQC) events. Note the Sigma scale at the bottom of the diagram. To apply, determine Sigma-metric, locate on the Sigma scale, identify control rules, total number of control measurements (N), and frequency of SQC events specified as run size. CV - coefficient of variation. TEa - total allowable error.

Other authors adapt Westgard’s rules to the size of the series according to the Sigma level and error detection probabilities (*11*, *15*, *16*) (Figure 1).

### Exponentially weighted moving average

In order to improve the detection of small trends, in 1959 Roberts proposed the exponentially weighted moving average (EWMA) based on Bayesian statistics (*17*, *18*). The principle of EWMA is simple: each new IQC value is weighted by the previous values. It is a method of smoothing new results obtained by an exponentially weighted moving average, *i.e.* it favours the most recent points at the expense of the old ones. In practice, this allows an earlier detection of small deviations and a better detection of systematic errors (*19*, *20*).

## Changes in reagent and internal quality control lot

See risks 1 and 2

### Standard requirements of the ISO 15189:2012 (5.3.2.3)

The ISO 15189:2012 (5.3.2.3) - Acceptance test: “Each new formulation of examination kits with changes in reagents or procedure, or a new lot or shipment, shall be verified for performance before use in examinations.”

To verify the performance of reagents and consumables, the laboratory establishes an acceptance strategy based on a risk analysis. For example, the use of supplier data, certificates of conformity and the strategy for implementing quality controls (*4*).

For each change of control lot (IQC), the laboratory must ensure that it plans to calculate new target values and interpretation thresholds. These are determined according to preliminary tests defined by the laboratory, depending on the specificity of the test and the validity period of the batch. During this period, the conformity of the technique is ensured by the current control lot.

The average of the results will determine the initial target value. The thresholds of the new control charts will be readjusted if necessary.

The number of preliminary determinations will be adapted to the duration of use of the batch of IQCs (very short period of 1 to 2 days for haematology for example, to a longer period in the case of a batch of IQCs of one year (some IQCs used in haemostasis or biochemistry for example)). Another approach is to use associated statistical tests based on Bayes’ theorem (*18*).

### Acceptance testing

The objective of acceptance testing is to ensure that the product (reagent, IQC, consumable) meets the laboratory’s needs before authorizing its use, and early enough to be able to order a new batch or shipment (or to organize a backup plan, or subcontracting) to avoid any production interruption that may lead to any risk for the patients in the event of critical test. The strategy is to be defined for each analyte based on the available data and identified risks (Table 12).

**Table 12:** Control panel

Recommended practice	The choice of rules, the number of IQCs and the size of the series are based on the calculation of the Sigma level or any other means of assessing robustness.
Acceptable practice	Use rules 1-3S, 2-2S (intra- and inter-series) and R4S to detect errors (random and systematic). In the case of a floating average IQC value, use the drift detection rules (7x, 10x, ...). Documented control charts used to detect method drift or offset.
Unacceptable practice	Undocumented and misdirected control charts that do not allow following the drifts or the movement of the method.
IQC – internal quality control.	

#### New batch of internal quality control materials

The recommendations in the literature are (*10*, *13*):

determination of the target value by the laboratory (10 measurements over 10 days)
determination of the standard deviation by the laboratory (20 measurements)
the calculation of limits from the laboratory mean and standard deviation (or the use of a standard deviation defined by the laboratory based on its experience).

The data will need to be updated after a few weeks to obtain values that take into account greater real variability, such as maintenance, calibrations, *etc.* (Table 13).

**Table 13:** New batch of IQC

Recommended practice	Overlap period for each IQC batch change. If possible, several days (depending on how long the batch has been used): 2 days in haematology, 10 days if the batch is used over a long period of time). Consultation of peer averages if available (external IQC).
Acceptable practice	Minimum overlap period of one day and use of previous CVs (may nevertheless deviate significantly from the objective).
Unacceptable practice	No evaluation of a new batch of practice IQC before use with risk of impact on the patient in case of simultaneous deterioration of the reagent and the IQC.
IQC – internal quality control.	

#### New reagent batch

The risk analysis must be evaluated during method verification/validation: define the impact of the product (diluent, reagent, calibrator) (*21*, *22*). Is the product critical and how robust is it?

The analysis of “fresh” patient samples is the reference method but other methods can be used (average patients, pools, *etc*.); isolated measurement of an IQC material to validate a new batch of reagent is not recommended (*11*).

Nevertheless, for some substrate tests (blood sugar, cholesterol, *etc.*), the commutability of IQCs seems sufficient. In hormonology, lot-to-lot effects are common and IQC comparison is not always relevant. For tumour markers, the IQC is often non-commutable and running “fresh” samples from patients is strongly recommended (Table 14).

**Table 14:** New batch of reagents

Recommended practice	Evaluation of the new reagent batch before production. New reagent lot evaluated from patient samples (at least 3) or other argumentative strategy (21). Evaluation of the new reagent lot with “fresh” patient samples only for tests with uncertain IQC commutability (mainly tumour markers, hormonology).
Acceptable practice	Evaluation of the new reagent at the start of production, but risk of disruption of activity and delay of results, with possible loss of opportunity for the patient (for some tests) and the importance of support to control this risk.
Unacceptable practice	No traceability of exchanges of reactive practice batches. No evaluation of a new reagent lot in a series between 2 IQCs (calibration validated without going through all IQC levels). No backup solution for critical analytes (volume, sample storage time).
IQC – internal quality control.	

#### New reagent formulation, new reference?

In the case of a new reagent formulation (and a new reference on the supplier’s site), the laboratory must carry out an impact study, based on the supplier’s documentation:

a simple bibliographical study, which concludes to the absence of impact or to a purely documentary impact: change of packaging, change of storage mode without impact on the method, *etc.*
check changes only: comparison with previous results if a new calibrator is assigned. Study of the impact on accuracy or precision and possibly on reference values.
complete verification of the method in case of major changes.

## Trend detection, quality indicators

### Trend detection

#### See risk 8

The ISO 15189:2012 standard 5.6.2.3 recommends that “Quality control data should be reviewed at regular intervals to detect trends in examination performance that may indicate defect in the examination system. When such trends are identified, preventive measures are taken and recorded”.

Summary: what are the good practices for the calculation of the laboratory CV (acceptable limits)? The principle of quality control using Levey-Jennings charts is to determine whether a value belongs to the “usual” population or to a different population (shifted average and/or higher CV) that has arisen because of an analytical malfunction. Statistical parameter estimation (mean, standard deviation or CV) is the process of estimating population parameters from a statistical sample taken from the population. If the statistical sample contains values from two different populations, the calculation no longer makes sense. The “usual” dispersion of a method corresponds to the causes qualified as “common”; the IQC values to be maintained are those that reflect the performance of the method.

If an analytical malfunction is detected by out-of-control values (violation of rules 1-3S, 2-2S, R-4S), an “exceptional” cause is present and therefore the values obtained under these conditions do not belong to the “usual” population. Patient results are not released until the disorder is corrected. Therefore, it seems logical and consistent not to include these values in the calculation of a CV that should represent the “usual” operation of the laboratory.

On the other hand, if the re-running IQC provides values within acceptable limits, it can be concluded that the previously uncontrolled value is part of the “usual” population. This is the first type of risk (3 *per* thousand in the case of rule 1-3S). It is then recommended to include this value in the CV calculation. The IQC results to be included in the calculation of the laboratory’s intermediate CV are those that reflect the actual performance of the method and are consistent with patient results. If the biologist decides to report the patient’s results, for example after evaluating the patient against the total allowable error, the IQC results must be included, otherwise, when the run is rejected, the IQC results will be excluded. This methodology has a double coherence: statistical and in relation to analytical goals.

Major errors (inversion of IQC levels, end of vial, *etc.*) which do not represent the real dispersion of the method are therefore not taken into account. If the method is changed (change of reagent batch, recalibration, *etc.*), it makes sense to exclude non-compliant IQC points from the CV calculation (data before calibration or vial change).

On the other hand, if nothing is changed in the method (the same IQC is used), there is no justification to exclude these points.

The main trend to be observed is the increasing dispersion of the method with the CV. The follow-up of CVs and compliance with laboratory specifications is recommended. This check must be carried out regularly (depending on the robustness of the method), and at least quarterly. If several analysers are used to perform the same tests, it is recommended to compare the CVs of the different analyser systems.

The objective of trend analyses is to identify any drift in the analysis system earlier and to put in place the necessary preventive actions for implementation if necessary (random and systematic error monitoring):

an increase in the dispersion of the method (random error drift) can be objectified by regular monitoring of the method (frequency to be adapted for each review according to robustness, clinical significance, frequency of performance, *etc.*). For routine examinations, regular monitoring of the CV is recommended with comparison with the laboratory specifications, at an appropriate frequency, usually monthly depending on the examinations, in order to be able to act quickly if necessary;
an increase in bias (or bias of trueness) can be assessed by an external comparison of the IQC results (regular monitoring of the Z-score or standard deviation index) (Table 15).

**Table 15:** Trend detection

Recommended practice	Calculation of the CV from all the IQC values corresponding to the results reported by the patients, excluding major errors (CV operating rules defined by the laboratory). Regular monitoring of the CV (monthly, quarterly) according to the methods (and their robustness). Define CV ranges with arguments (national or international publications). Comparison with peer group CVs as part of the external evaluation of the IQC.
Acceptable practice	Regular monitoring of CVs (quarterly) but risk of greater impact in case of significant CV drift.
Unacceptable practice	Exclude IQC values outside the range without investigation, argumentation, and traceability. Frequency of CV follow-up (higher than quarterly follow-up) not supported by arguments. No definition of acceptable CV ranges. No impact assessment when CV ranges are exceeded.
CV – coefficient of variation. IQC – internal quality control.

### Targeting internal quality control values

Each laboratory determines the target value, which is the average of the values obtained during the probationary period. This value is used as the average value for the control chart. When using an inspection sample lot, the target value can be readjusted if necessary. The target value is calculated over a sufficiently long period of time to be meaningful (*4*).

In the event of a shift of more than one standard deviation (unit of measurement of the control chart), the laboratory must retarget the mean to avoid the risk of false rejection.

In the case of compensation, the laboratory documents the retargeting or comparison of results with a peer group or with the EQA(s).

Any “retargeting” is documented and preceded by a study of the potential sources of variation (calibration, change of reagent batch, maintenance, *etc.*). In addition, the laboratory must ensure that there are no errors in trueness (with the peer group average) or accuracy (with the EQAs) (Table 16).

**Table 16:** Targeting IQC values

Recommended practice	Regular analysis of control charts practice (weekly). In the case of compensation, the laboratory documents the retargeting or comparison of results with a peer group or with the EQA(s).
Acceptable practice	Shift in the mean (more than one standard deviation) that may result in false rejection without retargeting. Shift that can lead to false acceptance but without any clinical impact. Control cards with floating average and rigorous monitoring of any discrepancy (activation of the corresponding rules (*e.g.* 10x, *etc*.).
Unacceptable practice	Re-screening without investigation or argument (except in the case of floating average control charts).
IQC – internal quality control. EQA – external quality assessment.	

### Additional means for monitoring performance

These means can complement the strategies methods, in addition to the IQCs and external evaluation of the IQC and EQAs.

They are not mandatory but may provide additional information if used correctly.

#### Patient average

Patient mean monitoring (called XBar-M in haematology) can be a relevant complementary means of early detection of drift or shift in an analytical system (*23*, *24*). This method has the advantage of evaluating the offset of the method with respect to a human matrix and allows compensating for a commutability problem or a deterioration of the IQC. Tracking of patient averages is not relevant for all tests (infectious serology, tumour markers, *etc.*).

When using patient averaging, particular attention should be paid to:

population size (which will enable the average to be calculated and the value to be plotted on the control chart to be defined) and the exclusion of certain critical services (dialysis, intensive care): indicator applicable only to a stable population
the acceptable limits defined by the laboratory, which must be, at a minimum, comparable to those of the IQCs (in terms of CVs, even if statistically not the same CV associated with a population)
measures to be taken in the event of an alert (Table 17).

**Table 17:** Patient average

Recommended practice	Follow-up of the patient average after deletion of pathological values and/or exclusion of non-representative patients/services (intensive care, dialysis, *etc*.). Statistical definition of acceptable limits (standard deviation of results/root N, where N is the size of the series for which the mean is calculated). Possible different weighting of the last point(s) to increase sensitivity.
Acceptable practice	Acceptable X-bar limits comparable to acceptable IQC limits.
Unacceptable practice	Acceptable X-bar limits well above acceptable IQC limits and use of the X-bar. To override invalid IQCs (the X-bar cannot be used for this purpose alone but is one of the elements to be considered in the data analysis).
IQC – internal quality control.

#### Retesting of patient samples

The analysis of patient samples several times a day (within the limit of analyte stability) can be used as a performance control (“patient reference sample” or “patient QC”) with an acceptance criterion of 2.8 times the standard deviation of the method (*25*). This compensates for the non-commutability of some IQCs and may also make it possible to study it (Table 18).

**Table 18:** Reanalysis of patient samples

Recommended practice	Selection of patient samples at different concentration levels. Relevant validation of the new test (2.8 times the actual standard deviation of the method). Monitored as an indicator (percentage of releases).
Acceptable practice	Only samples from normal patients are used. Use only in case of new test criteria established by the laboratory (monitoring as an indicator).
Unacceptable practice	No formal validation criteria for new tests as part of performance monitoring. Exclusive use of this system without IQC testing.
IQC – internal quality control.	

#### Patient pool

The use of a pool of samples from patients may be a solution chosen by the laboratory. This may be appropriate, particularly in the absence of IQCs and/or as a complement to IQCs, to explore commutability (tumour markers, hormonology, *etc.*). The stability of this pool of samples, often frozen, must be explored and documented (Table 19).

**Table 19:** Patient pool

Recommended practice	Test of pool stability (proof of storage control). Target and acceptable limits defined as an IQC (see above). To be used when changing reagent batches.
Acceptable practice	Stability is not demonstrated, but performance is controlled and comparable to IQCs.
Unacceptable practice	Acceptable limits not defined. Use of an unstable pool due to improper storage
IQC – internal quality control.	

#### “Sentinel” tests

On a multi-test instrument, some tests are more sensitive than others, due to the different components/features of the instrument (low volume pipetting, specific wavelength, reaction time, *etc.*).

A suitable schedule of IQCs or patient samples for these tests allows monitoring of all other methods (especially if they are robust). Control of the risk linked to internal maintenance or at the manufacturer’s (after-sales service) can be based on a panel of these “sentinel tests” in order to quickly check that maintenance has no impact on the various components of the analyser. The strategy shall be justified, in particular based on the data provided by the manufacturer and the assessment of the robustness of the method.

In the event of “major” maintenance requiring calibration, this strategy cannot be adopted (Table 20).

**Table 20:** Sentinel tests

Recommended practice	Definition of “sentinel tests” (based on robustness, analyser configuration, *etc*.) using the manufacturer’s instructions (most reliable source of information on this subject). Definition of a strategy for conducting IQC or patient samples for these “sentinel tests”. Monitoring of these performance indicators.
Acceptable practice	Implemented but lack of monitoring of the indicator.
Unacceptable practice	Error in the choice of “sentinel tests” (which are not the most sensitive).
IQC – internal quality control.	

## Post-IQC impact assessment, end of run outside acceptable limits

### Regulatory requirements ISO 15189:2012 (5.6.2.3)

Where quality control rules are violated and indicate that the examination results are likely to contain clinically significant errors, the results are rejected and the relevant patient samples are re-examined after the error condition has been corrected and performance in accordance with specifications has been verified (*13*). The laboratory must also evaluate the results of patient specimens that have been examined after the last successful quality control.

Document SH Ref 02 specifies that alarm and action thresholds must be defined. In the case of non-compliant IQCs, the laboratory must assess the impact on the results obtained since the previous compliant IQC (*4*).

### Provisions

The laboratory must have a study of the potential impact in the event of a failure affecting patient samples (Table 21).

**Table 21:** Post-IQC impact assessment, end of run outside acceptable limits

Recommended practice	Clear provisions: understandable for the staff responsible for applying them (adapted to the quality management system). Complete provisions: any type of method (quantitative/qualitative, automated/manual...), analyte (variable criticality), activity (continuous/discontinuous). Adaptable provisions: to all situations encountered.
Acceptable practice	No precise provisions, but correct practices and an impact study if necessary.
Unacceptable practice	No provision and no impact study in case of non-compliance with the IQC.
IQC – internal quality control.	

### Reason for method drift

The laboratory must verify that the non-compliant IQC reflects a malfunction in the analytical system. First, exclude the IQC’s liability by verifying that a freshly prepared control is non-compliant or that retesting fresh samples from patients proves that the results are non-compliant (otherwise there is a risk of false rejection with adverse consequences for the laboratory (in terms of time and cost)).

The laboratory must then investigate the causes of the problem and determine the extent of the problem:

which analytes are involved and at what levels?
IQC analysis (all levels) or re-analysis of patient samples;
what’s the probable cause? Explicit alarm or not?
how long has this been a problem? IQC examination, alarms, floating average calculation to look for possible drift.

The scope analysis should define the magnitude of the problem and identify the list of samples that could be affected.

### The strategy of the impact study

Step 1: The choice of samples to be re-analysed depends on the number of samples potentially affected. Use the results of re-tested samples to verify that analytical acceptance limits are below 2.8 CV (*25*).

Step 2: In the absence of analytical agreement, define clinical acceptance limits:

the analytical objectives of the Milan Consensus apply (despite data from a few clinical studies). In practice, the total error of desirable biological variation can be used (*26*, *27*).
EQA acceptance limits can also be used (they define a clinical impact) and are generally relevant.

If the limits of clinical acceptance are exceeded, a reminder of the test report is necessary if it has already been published (modification of test reports according to 5.9.3 of standard ISO15189:2012). The SH Ref 02 states that erroneous test reports are replaced, and discussion with the clinician is very important (Table 22).

**Table 22:** Impact assessment strategy

Recommended practice	Complete list of affected samples. Evaluation of analytical variations. Clinical impact study (in case of analytical discrepancy). If warranted, provide updated reports to prescribers and patients. Extensive traceability of all stages of the impact study in the event of non-compliance.
Acceptable practice	No distinction between analytical and clinical discrepancies and excessive patient recall. Non-systematic traceability of all steps of the impact study, but at least traceability of recovery calculations and report modifications.
Unacceptable practice	Missing some potentially impacted files. The inability to identify analytical and especially clinical discrepancies. No transmission of modified laboratory test reports and no argumentation
IQC – internal quality control.	

### Analyser comparability

See risk 8

If several analysis systems are used to perform the same tests in the laboratory, comparability of the results provided by the different systems must be ensured. Point of care testing (POCT) devices are also concerned by this comparability study.

### Frequency of monitoring

The laboratory must define a control frequency. There is no opposable recommendation for this frequency, but the laboratory must prove it on the basis of its risk analysis taking into account the number of tests, the robustness of the methods, the consequences of a drift of one of the systems, other means put in place (IQC with common acceptable limits, comparison of floating averages, *etc*.) (Table 23).

**Table 23:** Multi-analyser comparability: frequency of monitoring

Recommended practice	Regular comparison based on the robustness of methods and risk analysis. If the IQC deviates by more than one standard deviation from the mean of all instruments in the same analyser (if the laboratory uses the same mean and standard deviation for all instruments). If the patient’s average (X bar) changes on an analyser (several alarms). In the event of a batch change of a sensitive reagent. In the event of an isolated failure of an EQA on an analyser. In case of major corrective maintenance.
Acceptable practice	Comparisons at a defined and acceptable frequency with intervention and correction of the problem in case of drift, but without explaining the reasons for the action or providing a specific risk analysis.
Unacceptable practice	No comparison. Frequency not suitable for rapid detection of system drift.
IQC – internal quality control. EQA – external quality assessment.	

### Possible materials to be used

Several possibilities are available to ensure this analytical comparability: IQC, fresh patient samples, pools of stored samples, EQA samples, statistical studies of results (*e.g.* patient averages), *etc.* (Table 24).

**Table 24:** Multi-analyser comparability: possible materials to be used

Recommended practice	Reanalysis of fresh patient samples on the different systems at a frequency defined by the laboratory and statistical validation according to standard ISO 5725-6:1994 (24).
Acceptable practice	IQC within acceptable limits for all systems EQA tested on all analysers with laboratory interpretation by the laboratory (statistical evaluation based on ISO 5725-6:1994) or by the EQA providers (provided that the frequency of these EQAs is sufficient)
Unacceptable practice	Different acceptable limits on analysers. Compare only by looking at the average of normal patients (unable to quickly detect a discrepancy in one of the systems).
IQC – internal quality control. EQA – external quality assessment.	

### Indicators

In order to quickly detect system drift, the laboratory can also use several other indicators: percentage of IQCs rejected *per* system, monitoring of CVs of each system, CV ratio < 2 (expert proposal) between different systems, percentage of rejections as compared to the total number of data, number of EQA failures, percentage of rejections of patients re-tested (re-test criteria not met), percentage of qualitative haematology alarms *per* analyser. These indicators are early warnings that should lead to an investigation.

### Assessment of clinical impact

In the event of an analytical difference, the laboratory must define the clinical impact.

The final decision to recall the final report depends on the critical difference between 2 results, *i.e.* the definition of clinical impact.

The different formulas and concepts that the laboratory can use are summarized in Table 25.

**Table 25:** Multi-analyser comparability: formulas and concepts for clinical impact assessment

**Name**	**Abbreviation**	**Formula**	**CV_A_**	**Bias**	**CV_I_**	**CV_B_**	**Clinical data**
Bias (CLSI EP31)	Bias	0.33 x CV_I_	No	No	Yes	No	No
Total allowable error	TE_A_	(1.65 x I) + Bias	No	No	Yes	Yes	No
Reference change value	RCV	√2 x z x (CV_A_^2^ + CV_I_^2^)^1/2^	Yes	No	Yes	No	No
Total value of change	TCL	((2.77 x (CV_A_)^2^) + (0.5 x CV_I_)^2^)^1/2^	Yes	No	Yes	No	No
Measurement uncertainty	MU	2 x (CV_A_^2^ + Bias^2^)^1/2^	Yes	Yes	No	No	No
Clinical Outcomes	CO	Physician experience	No	No	No	No	Yes
Score Z or SDI	EQA providers	Bias/CV	Yes	Yes	No	No	No
CV_A_ – analytical coefficient of variation. I – imprecision. CV_I_ – Intra-individual biological variation. CV_B_ – Inter-individual biological variation. SDI – standard deviation index.							

Remark: The acceptable limits defined by the EQA or proficiency testing (PT) providers are specified according to the Milan hierarchy and depend on the analyte: they may be based on the experience of the EQA/PT providers, the total error allowed, *etc.* (Table 26) (*26*).

**Table 26:** Multi-analysers comparability: clinical impact assessment

Recommended practice	Use of the Milan hierarchy (clinical impact, biological variations, state of the art for each analyte).
Acceptable practice	Use of one of the acceptable criteria but without thought and reasoning.
Unacceptable practice	No distinction between analytical and clinical discrepancies and recall of missing patients.

## External comparison of internal quality controls

The external comparison of the IQCs is a complementary tool that allows to (*28*, *29*):

check the trueness of the analytical method against the peer mean and the peer standard deviation or CV
ensure retargeting in the event of internal change (or to put in place a control chart in the absence of a “probationary period”)
obtain method specifications (CV, long-term CV, bias, uncertainty, *etc.*) (Table 27).

**Table 27:** External comparison of IQCs

Recommended practice	Evaluation of the truthfulness of the peer group average. Monitoring of CV (indicator) and SD_I_ ratios on a regular basis (monthly or batch): a CV_I_ greater than 1 reflects a performance that needs to be monitored (28). Use SD_I_ values (Z-score) greater than 2 as an alarm. A Z-score higher than 3 indicates a difference with the other participants (caution: the Z-score depends on the dispersion of the results, to be interpreted according to the population of participants).
Acceptable practice	Temporary use of peer average as internal laboratory average. Temporary use of peer standard deviation to calculate acceptable limits for laboratories.
Unacceptable practice	Use of IQCs with peer mean and standard. Incorrect interpretation of data (SD_I_ and CV_I_). Absence of reaction in the event of lasting performance degradation, without justification.
CV – coefficient of variation. SD_I_ – standard deviation. IQC – internal quality control. CV_I_ – intra-individual biological variation.

## External quality assessment

### Selecting an external quality assessment

The criteria for selecting an EQA are presented in the Table 28 (recommendations). Commutability is desirable, but the information on these data is inconclusive (*30*-*32*). The laboratory can analyse EQA reports to assess these data (*e.g.* by comparing the differences between the different methods depending on the nature of the control samples).

**Table 28:** Choosing an EQA

Recommended practice	EQA body accredited according to ISO/IEC 17043 (an accredited EQA body is considered impartial and independent) or for non-accredited EQA bodies, independence from suppliers and participating laboratories. The EQA body undertakes to carry out reactive vigilance declarations in the event of anomalies observed. Availability and cooperation with expert medical biologists. Adequacy with the needs of the laboratory (relevance, frequency and alternation of the proposed levels). Switching capability certified by the EQA body (data available later) or information on this switching capability. Relevance of evaluation: number of participants in peer groups or any methods and statistical analysis. Clarity of reports. Discussion of clinical cases
Acceptable practice	EQA bodies present on the annual report of external quality assessment bodies published by the French National Agency for the Safety of Medicines and Health Products. If the EQA body is not accredited, the laboratory will verify that the EQA body is working according to ISO/IEC 17043 (independence or implementation of measures to ensure no conflicts of interest with suppliers of analytical/reagent systems, non-disclosure of assigned value before the closing date, no subcontracting of assessment and planning, conformity of report content, *etc.*)
Unacceptable practice	Direct dependency on a supplier. Inappropriate frequency and levels. Unclear or missing reports (different language, content not conforming to ISO/IEC 17043 standard).
EQA - external quality assessment.	

### Participation in external quality assessment

Laboratories carry out an EQA of each analytical system they use (*33*).

The laboratory should analyse its EQA as a patient (once) and should not repeat the measurement unless required by these provisions (systematic re-test rules).

When measuring EQAs with multiple analysers, in case of discrepancies, the laboratory will report the EQAS result obtained for each analyser.

### Acceptable limits and interpretation of results

Three types of objectives can be used for evaluations: average of all method values, peer group average and value obtained with the reference method (if possible).

For each assessment, acceptable limits are defined by the EQA body (clinical needs, state of the art and biological variations) (*33*, *34*).

Each organizing body defines its own acceptable limits based on literature data (Table 29).

**Table 29:** EQA: Acceptable limits and interpretation of results

Recommended practice	Knowledge of acceptable limits and their origin (comparison with acceptable limits chosen by the laboratory (see above)). Interpretation in relation to the results of other participants (z-score): beware of the limits of the z-score (widely scattered results for the method will give a false z-score and *vice versa*). Interpretation in relation to acceptable limits (leading to clinical impact). Responsiveness in the event of non-compliance with the EQA: search for causes, impact on the patient, *etc.* (action recorded with systematic impact study).
Acceptable practice	Use of z-score and systematic impact assessment if z-score > 3. If the CV of the method is increased (and there are no acceptable limits available), analyse the clinical impact of the result even if the z-score is less than 3. Use of the rating according to the recommendations of the organizing body.
Unacceptable practice	No impact assessment in case of non- compliance of the EQA. If reference values against a method-independent threshold, interpretation only for peer groups (subject to control commutability).
EQA - external quality assessment. CV – coefficient of variation.	

### In the absence of an available external quality assessment

In rare cases where no EQA is available (a rare situation for routine testing), the laboratory must demonstrate the accuracy of the results provided (Table 30).

**Table 30:** Absence of EQA: guidelines

Recommended practice	Use of CRMs (certified reference materials) if they exist and are reasonably accessible. Compare with at least one other laboratory. Externalisation of the IQC.
Acceptable practice	Use of samples stored in the laboratory (subject to stability) and at least one inter-laboratory comparison.
Unacceptable practice	No inter-laboratory comparison.
CRM – certified reference material. IQC – internal quality control. EQA - external quality assessment	

### Interpretation of measurement uncertainties and their calculation

Care should be taken not to confuse total error with measurement uncertainty (MU): total error is the difference between the measured value and the actual value, while uncertainty is the quantification of doubt around the measured result (*35*).

Measurement uncertainty is particularly important when interpreting the result in relation to a decision threshold with consequences for the medical impact of patient care (haemoglobin level and transfusion, drug dosage and dosage adjustment, carbohydrate transferrin deficient (CDT) and driver’s license, *etc.*).

In practice, the calculation of uncertainty is based on the quadratic combination of 2 terms: imprecision and bias. For the bias, one can use the mean bias (with the standard deviation of the bias) or the maximum bias (*35*).

The MU shall be evaluated according to the clinical interpretation required by clinicians, taking into account the clinical needs as referred to in ISO 15189:2012 (*3*).

If the two components of the measurement uncertainty (standard deviation and bias), monitored regularly have not changed, the monitoring can be spaced out. Nevertheless, the EQAs bodies that provide an estimate of the MU carry out an annual review with, above all, a comparison of all the participating laboratories. Finally, the choice of performance requirements is difficult: the total error is not rigorously statistically comparable and there is little other recent data in the literature. The monitoring of measurement uncertainties remains an internal tool based on the monitoring of the analytical performance of the parameters in practice (Table 31) (*36*, *37*).

**Table 31:** Interpretation of measurements uncertainties and their calculation

Recommended practice	Calculation with recognized components (accuracy and precision) and comparison with limits calculated according to the same formulae. Define performance requirements for clinicians in interpreting results. Compare the results of the calculated uncertainty of measurement with other laboratories. Periodically evaluate the accuracy or correctness of the method change and recalculate the MU if necessary, at the medical level of the decision. Use MU around the medical decision threshold for data interpretation. All biologists interpreting the results must be aware of this information and make it available to prescribers.
Acceptable practice	A calculation made by an organizing body is acceptable. As part of the estimation of the MU, a provisional comparison with the total error is acceptable, pending a calculation taking into account the quadratic propagation of the imprecision and trueness components.
Unacceptable practice	No calculation of the MU and/or incorrect calculation formula.
MU –measurement uncertainty.	

## Point of care testing and quality controls

The risk analysis must be carried out specifically for these tests. In particular, take into account the volume of tests carried out and the use made of the results.

If the IQC can be used with POCT the recommendations are similar (Table 32).

**Table 32:** Simplified unit testing, off-site laboratory testing: associated quality controls

Recommended practice	The controls included in the kit are carried out at a defined frequency following a risk analysis that has helped define the laboratory’s strategy: if positive and negative controls are provided by the manufacturer, it is recommended that they be tested at a minimum at each new shipment and batch change. In case of absence of positive intra-case control, an IQC must be set up and tested at the same frequency. An IQC that “mimics” the sample of patient must be used preferably. The laboratory may include other entry elements in its strategy: newly authorized operator, batch reaching the end of its shelf life, *etc.* Participation in EQA are appropriate, and if not, comparison with the results of the same non SUT methods and not POCT. Checking the comparability of results when changing batches.
Acceptable practice	IQC for each new acceptable shipment and batch change
Unacceptable practice	No EQA. No other IQC than sample migration control (for the simplified unit test).
IQC – internal quality control. EQA – external quality assessment. SUT – Simplified unit testing. POCT – Point of care testing.	

## Conclusion

This document is available free of charge and the authors have already received about a hundred suggestions for improvement from all types of French laboratories (University Hospitals (CHU), General hospital (CHG), reference laboratories, private laboratories). This document will be regularly revised to adapt to scientific and technical developments in test methods.

This document is intended to define a quality control strategy (IQC and EQA) based on a risk analysis and including the process of validation and/or continuous verification of the method. This strategy associated to statistical methods will be a help for the laboratories to provide expected reliable results meeting the needs for patient care.
